# Intelligent Microfluidics for Plasma Separation: Integrating Computational Fluid Dynamics and Machine Learning for Optimized Microchannel Design

**DOI:** 10.3390/bios15020094

**Published:** 2025-02-06

**Authors:** Kavita Manekar, Manish L. Bhaiyya, Meghana A. Hasamnis, Madhusudan B. Kulkarni

**Affiliations:** 1 Department of Electronics Engineering, Shri. Ramdeobaba College of Engineering and Management, Nagpur 440013, MH, India; 2 Department of Electronics and Communication Engineering, Manipal Institute of Technology, Manipal Academy of Higher Education (MAHE), Manipal 576104, KA, India

**Keywords:** blood plasma separation, packed cell volume (PCV), computational fluid dynamics (CFD), intelligent microfluidics, machine learning, healthcare application

## Abstract

Efficient separation of blood plasma and Packed Cell Volume (PCV) is vital for rapid blood sensing and early disease detection, especially in point-of-care and resource-limited environments. Conventional centrifugation methods for separation are resource-intensive, time-consuming, and off-chip, necessitating innovative alternatives. This study introduces “Intelligent Microfluidics”, an ML-integrated microfluidic platform designed to optimize plasma separation through computational fluid dynamics (CFD) simulations. The trifurcation microchannel, modeled using COMSOL Multiphysics, achieved plasma yields of 90–95% across varying inflow velocities (0.0001–0.05 m/s). The input fluid parameters mimic the blood viscosity and density used with appropriate boundary conditions and fluid dynamics to optimize the designed microchannels. Eight supervised ML algorithms, including Artificial Neural Networks (ANN) and k-Nearest Neighbors (KNN), were employed to predict key performance parameters, with ANN achieving the highest predictive accuracy (R^2^ = 0.97). Unlike traditional methods, this platform demonstrates scalability, portability, and rapid diagnostic potential, revolutionizing clinical workflows by enabling efficient plasma separation for real-time, point-of-care diagnostics. By incorporating a detailed comparative analysis with previous studies, including computational efficiency, our work underscores the superior performance of ML-enhanced microfluidic systems. The platform’s robust and adaptable design is particularly promising for healthcare applications in remote or resource-constrained settings where rapid and reliable diagnostic tools are urgently needed. This novel approach establishes a foundation for developing next-generation, portable diagnostic technologies tailored to clinical demands.

## 1. Introduction

Plasma separation from whole blood is an important stage, since 90% of diagnostic tests use blood plasma; therefore, blood plasma is stated to be the “liquid gold” of health diagnostics [1]. Separating and enriching blood cells can aid in the detection of various diseases, including cancer, HIV, and malaria [2]. Thus, implementing an enhanced technique for blood plasma and hematocrit (PCV) separation is essential in healthcare, since the two biomarkers (plasma and PCV) are helpful in the diagnosis of several diseases [3]. Blood, a viscous liquid consisting of various components such as red blood cells (erythrocytes), white blood cells (leukocytes), platelets, plasma, and assorted substances, holds significant importance in biomedical and biomechanical domains [4]. The entirety of blood comprises 55% plasma and 45% red blood cells, also known as packed cell volume [5] (PCV) or “hematocrit”, which is the measure of the ratio of the volume occupied by the RBCs to the volume of whole blood in a sample [6].

**Figure 1:**
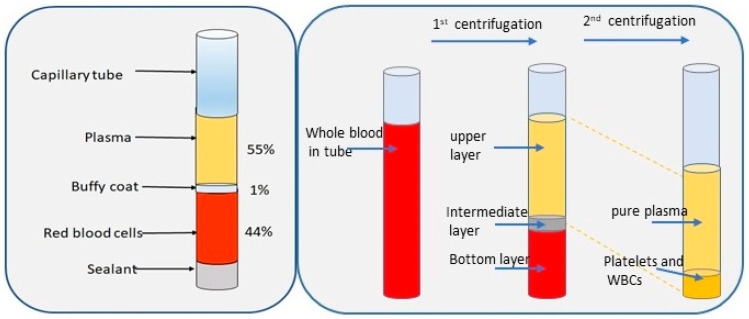
Representation of blood composition and centrifugation process.

Figure 1 presents the whole blood composition and the two-step centrifugation process. The centrifugal method is a traditional separation technique that relies on the viscosity of whole blood [7]. Additionally, filtration is another commonly employed method for separating plasma from blood, based on particle size. Traditional separation techniques, such as centrifugation, are resource-intensive, time-consuming, and reliant on bulky equipment [8]. These limitations make conventional methods unsuitable for rapid or on-site diagnostics, especially in resource-limited or remote settings where healthcare access is constrained [9]. Thus, there is a pressing need for alternative, efficient, and portable plasma separation technologies.

Microfluidic platforms have emerged as promising solutions for addressing these challenges. By leveraging advanced principles of fluid mechanics, microfluidic systems facilitate the miniaturization of diagnostic processes while maintaining accuracy and reliability [10]. These platforms offer several advantages, including reduced sample volume requirements, precise control over fluid dynamics, portability, and ease of integration with biosensors [11,12]. The different microfluidic platforms are capillary-driven, pressure-driven, centrifugal-driven, electrokinetic-driven, droplet driven, etc. [13]. The microfluidic blood plasma separation methods are classified as active and passive methods [5]. Passive separation techniques are preferred over active methods due to their tendency to circumvent design intricacies, their ease of integration with biosensors, and their cost-effectiveness [5,14]. The passive techniques offer various basic geometries such as Y channels, T channels, constriction–expansion, and spiral designs [15]. Out of these passive techniques, the branched bifurcation is an efficient which utilizes biophysical effects and depends on hydrodynamic separation [16]. This technique controls flow rates to segregate suspended particles (PCV) in a fluid based on their size and the bifurcation principle [17]. Notably, suspended particles (RBCs) tend to migrate towards the branch with a higher flow rate [18], a phenomenon known as the Zweifach–Fung effect or the bifurcation law [13,19]. Viscosity is an important rheological property for modeling blood flow in separation channels [20]. Despite these benefits, designing microfluidic systems for plasma separation is often a resource-intensive and iterative process. This challenge arises due to the lack of predictive tools for optimizing microchannel performance, leading to extensive experimental prototyping.

The research gap in the integration of machine learning (ML) with microfluidic systems lies in the limited scalability and adaptability of existing ML models for diverse microfluidic applications. While ML significantly enhances device design, data analysis, and automation, challenges remain in achieving seamless integration with real-world workflows, such as cost-effective fabrication, robust data privacy frameworks, and compatibility with existing healthcare systems. Addressing these gaps could unlock the full potential of ML-enhanced microfluidics for precision diagnostics, personalized medicine, and health monitoring [21]. The combination of microfluidics with ML has facilitated the development of intelligent devices, allowing microfluidic systems to attain unprecedented capabilities that were previously unattainable [22]. Combining the advantages of microfluidics, such as high throughput, miniaturization of fluid flow, and controllability, with ML, which allows access to large complex datasets and enables surrogate modeling, has motivated to investigate the potential of microfluidics in real-time and high-dimensional spaces with less computational effort [23]. ML is a class of artificial intelligence-based method that enable algorithms to learn without direct programming [24]. The ML methods offer quicker execution times in contrast to statistical and stochastic models. Integrating various machine learning techniques holds promise for minimizing prediction errors [25,26].

The proposed platform employs a passive trifurcation microchannel design, modeled using CFD simulations in COMSOL Multiphysics. The design parameters, including channel geometry, inflow velocity, and Reynolds number, were optimized to achieve plasma yields of 90–95%. The simulations accounted for key fluid properties, such as viscosity and density, to mimic physiological conditions. Boundary conditions were carefully defined, including normal inflow velocities ranging from 0.0001 to 0.05 m/s and outlet pressures set to 0 Pa to simulate an open system. ML models were trained using simulation data to predict the output volume fraction (Φmax) and plasma yield. The ANN model, configured with default settings, demonstrated the highest predictive accuracy, followed by the KNN model. Performance metrics such as Mean Absolute Error (MAE), Mean Squared Error (MSE), Root Mean Squared Error (RMSE), and R^2^ were used to evaluate the models.

The integration of ML into microfluidic design has profound implications for clinical diagnostics. By enabling rapid, on-chip plasma separation, the proposed platform reduces reliance on bulky centrifugation equipment and accelerates diagnostic timelines. This is particularly valuable in point-of-care applications, where timely results can significantly impact patient outcomes.

Additionally, the platform’s adaptability and scalability make it suitable for diverse healthcare settings, from well-equipped laboratories to remote or resource-limited environments. By addressing the key limitations of traditional methods, this work lays the foundation for developing next-generation diagnostic tools that are portable, cost-effective, and accessible to a broader population. In summary, this study bridges critical gaps in microfluidic design by integrating ML and CFD to create a robust and efficient plasma separation platform. The contributions and insights presented here advance the state of the art and pave the way for future innovations in microfluidic diagnostics.

## 2. Design and Simulation of Proposed Trifurcation Plasma Separation Microchannel

**Figure 2:**
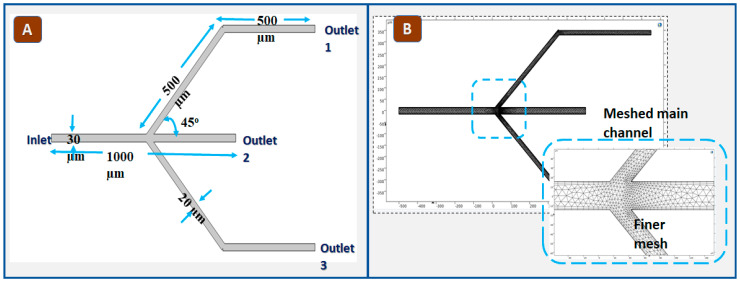
(**A**) The schematic of the trifurcation microchannel geometry model. (**B**) The meshed geometry of the model includes an inset zoom-in view, which illustrates the triangular-shaped mesh elements with higher density near the channel bifurcation.

The proposed work employs a passive branched microchannel design to separate plasma and packed cells encompassing four primary stages: 1. Microchannel geometry selection (varying channel width and height); 2. blood component properties selection (viscosity and density, as input fluid parameters); 3. application of the mixture model laminar f; and 4. a stationary study will be used to analyze simulation results. Adhering to the bifurcation principle, our microchannel was designed with a trifurcation pattern that has a single inlet as the main channel to input whole blood and three outlets. The setup includes a primary main channel measuring length (*l_m_*) = 1000 µm and height (*h_m_*) = 30 µm, it bifurcates into two identical sub-branches and forms the trifurcation microchannel. The two-dimensional design has the main channel comprising Inlet and Outlet 2 with the same geometry, as depicted in Figure 2A.

The channel height of the bifurcation sub-channel is reduced relative to the main channel in the geometric design. Thus, the secondary sub-channels, featuring bifurcating loops, are of a length (1000 µm) and height (*h_s_* = 20 µm) maintaining both the bifurcation angle of 45 degrees. The Fåhræus–Lindqvist effect plays an important role in understanding blood flow hemodynamics by causing red blood cells to migrate toward the center of the flow stream, resulting in a lower concentration of cells near the vessel walls [27]. Considering this effect, the design incorporates two bifurcations between the main channel and the sub-channels to direct plasma into the two sub-channels while keeping red blood cells in the main channel.

In this geometry, the main channel has high velocity and the sub-channel has low velocity [28]; hence, the RBCs are extracted from the main channel (outlet 2) and plasma is collected through the sub-channels (outlet 1 and 3). The density of the meshed geometry is modified according to the specific requirements of the design. This work includes physics-controlled mesh with finer element size. Figure 2B presents the bifurcation effect with finer mesh. The meshed geometry at channel bifurcation depicts the high density due to the bifurcation effect.

### 2.1. Methodology for Plasma Separation in Microchannel

In this work, blood is introduced as the input fluid to the microchannel, where it is treated as a mixture of liquid (plasma) and suspended particles (PCV or RBC). The study assumed blood behaves as a Newtonian fluid with laminar flow characteristics, simplifying computational requirements. While this assumption is valid for capillary-scale microchannels, it may not fully capture the shear-thinning behavior of blood at extremely low flow rates. Additionally, a two-dimensional geometry was used in the simulations to reduce computational complexity while preserving essential flow characteristics, although this approach may neglect certain three-dimensional effects. Limitations include the inability of the Newtonian model to represent blood’s shear-thinning properties accurately, particularly at lower shear rates, suggesting the need for non-Newtonian models in future studies. Thus, blood is modeled as a Newtonian fluid with incompressible and laminar flow characteristics within the branching microchannel. The fluid physics for the simulation is based on the mixture model laminar flow in COMSOL. Key properties of the blood include dynamic viscosity, measured in Pascal-seconds (PaS), and density, measured in kilograms per cubic meter (kg/m^3^). The whole blood has a shear-thinning behavior while flowing through capillaries [29]. Hence, in this work, the flow behavior of blood in the microchannel is described using the Power Law viscosity model (Equation (1)). Specifically, *μ*_app_ represents the apparent viscosity of the fluid, which is a function of the shear rate and the fluid’s rheological properties. The term *μ*_0_ represents the consistency index, and $\dot{\Upsilon }$ (gamma dot) is the shear rate, while *n* is the flow behavior index, which indicates whether the fluid is shear-thinning or shear-thickening.

(1) $${µ}_{app}={µ}_{0}{\dot{\Upsilon }}^{n-1}$$

The performance in a prescribed microchannel experiencing laminar flow is characterized using Flow rate, Reynold number, and Navier–Stokes equations for blood flow motion. These three constitutive fluid dynamic equations for modeling blood plasma microchannel in COMSOL are as follows:

The Reynolds number (Re):

(2) Re=ρvDhμ

Here, *ρ* is the density of the blood, *v* is the inlet velocity provided to the microchannel, *Dh* is a characteristic length, and μ is the dynamic viscosity of the blood.

The flow rate (Q):

Q = *whv* (3)

where *w* is the width of the channel, *lm* is for the main channel and *ls* is for the sub-channel, *h* is the height of the main or sub-channel (*hm* or *hs*), and v is the average velocity of the fluid.

The mixture model in COMSOL utilizes one set of Navier–Stokes, with Continuity and Momentum equations. The Navier–Stokes equations for incompressible blood flow, modeled as a mixture, are expressed in cartesian coordinates using COMSOL in Equations (4) and (5).

Continuity equation:

∇.*v* = 0 (4)

where *v* is the velocity vector field of the fluid. ∇⋅ is the divergence operator, which measures the rate of change of a vector field’s magnitude at a given point.

Momentum equation [30]:
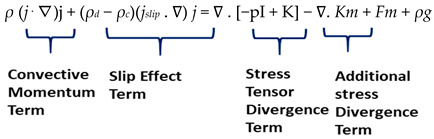
(5)

The first term represents the convective acceleration of the flow, *ρ* is the blood density in kg/m^3^, p is the pressure, I is the identity matrix, j is the velocity vector, and *Km*, *Fm*, *ρg*, and *F* represent external body forces that are additional stresses, which are negligible in this case. The slip velocity *j_slip_* given in slip effect term, the slip model selected is Schiller–Naumann.

The plasma yield is calculated to measure plasma purity by comparing the volume fraction of Packed Cells in the plasma channel to the overall volume fraction [31],

(6) $$Yield\;of\;Plasma=\frac{Volume\;fraction\;of\;RBCs\;in\;plasma\;channel}{Total\;RBCs\;phid(\phi d)}\times 100$$

The dispersed phase volume fraction *ϕ_d_* is a critical parameter in determining the effective viscosity of the suspension.

### 2.2. Simulations of Designed Microchannel

The design and modeling process were followed by more than 100 simulation sets with consistent microchannel geometry. The simulation for the designed structure involves using the ‘mixture model, laminar flow’ model in COMSOL. A 2D geometry is first chosen for the mixture model simulation, and a stationary study method is employed. In this scenario, the continuous phase is plasma, while the dispersed phase consists of packed cells within the mixture. The boundary conditions implemented in the model are as follows: The initial velocity field j in both the x and y directions is set to 0 m/s. The normal inflow velocity (*J*_0_) for each simulation was selected from 0.0001 to 0.05 m/s. to investigate its influence on the phase separation. The Schiller–Naumann slip model is applied to account for the relative motion between phases. Table 1 provides a comprehensive overview of all the constant input design values and the necessary boundary conditions for simulating the microchannel.

In the mixture viscosity model, since the dispersed phase is solid particles that are packed cell here, the Krieger-type viscosity model is selected by default. The expression for the mixture viscosity with Krieger type is

(7) $$µ=\mu_{c}{\left.\left(1-\frac{\phi_{d}}{\phi max}\right.\right)}^{-2\cdot 5\phi max}$$

For solid particles, the maximum packing concentration ϕmax, is 0.62. The *ϕ_d_* is the dispersed phase concentration and *μ_c_* is the viscosity of the continuous phase that is plasma. In this study, the input hematocrit (Hct) volume, denoted as *ϕ_d_*, is selected within the range of 15–46%.

**Figure 3:**
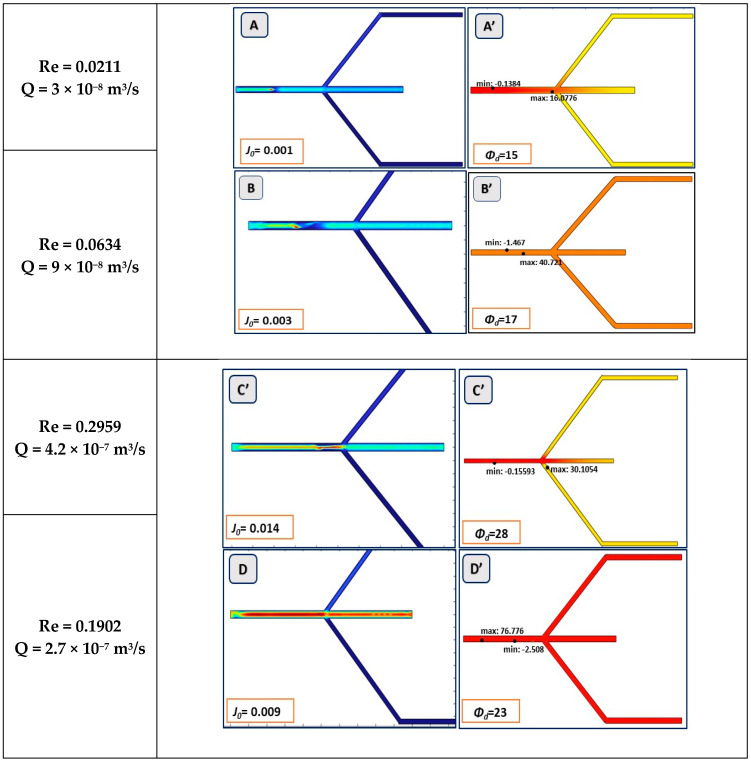

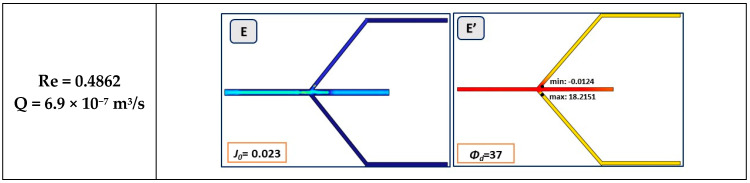
Simulation results in graphics. Images (**A**–**E**) depict output velocity profile in m/s along the channel length. Images (**A’**–**E’**) depict phase separation at different inlet velocities.

After applying the above-mentioned boundary conditions to the channel and the values of the other parameters, which can be obtained from past works in this field [32], the simulations were performed with a time-independent steady-state stationary solver in COMSOL. The simulation results for all runs are presented as graphics, with the title “Surface: velocity field, mixture (m/s)” for the velocity profile and “Dispersed phase volume fraction (1)” for the dispersed phase visuals. Figure 3A–E illustrates the flow profile of the mixture velocity distribution in the microchannel, with the five different values of inflow velocity (*J*_0_) in m/s and input dispersed phase volume (*Ø_d_*) in percentage. Additionally, Figure 3 illustrates the corresponding Reynolds number (Re) and flow rate (Q) in the main channel. The separation performance of microchannel at different inflow velocities can be observed in Figure 3A’–E’ in the form of dispersed phase volume fraction.

In the simulated mixture model, the flow behavior indicates that the velocity is lower near the side channels as the mixture passes through the bifurcation. The velocity profile graphics indicate that blood flow velocity is highest at the center of the main channel, which is the core area of the design. This increased velocity in the central region facilitates the rapid passage of Packed Cells (i.e., RBCs) through the main channel. The variation in inlet velocities results in different volumetric separations of whole blood in the main and sub-channels, as depicted in the graphics of Figure 3A’–E’. The difference in flow rate of the main channel and sub-channels was calculated using Equation (3). Figure 3B’,D’ shows no phase separation and illustrates the impact of varying inflow velocities on the volume distribution in the main and sub-channels. The trifurcation of hematocrit (RBCs) and plasma is observed in Figure 3C’,E’. Hence, the phase separation is observed for each simulation and data are generated for further process.

## 3. Trifurcation Microchannel Optimization for Plasma Separation Through ML

**Figure 4:**
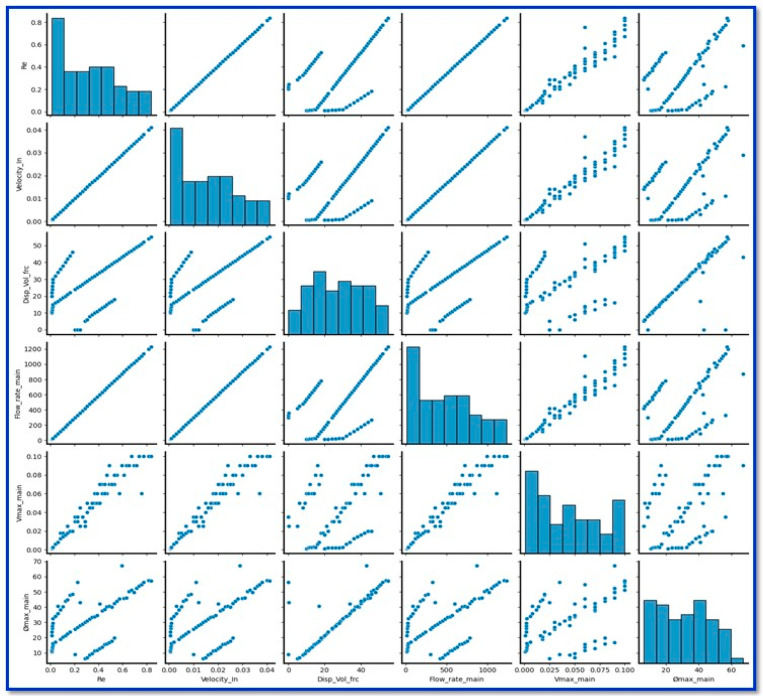
Correlation plots depicting effect of Reynolds no., inflow velocity and inlet volume fraction on flow rate, V_max_, and *ϕmax* in the micro channel.

The performance study for the proposed model is validated by comparing the plasma yield in sub-channels of the trifurcation microchannel. The output volume fraction is the key parameter to estimate plasma yield, therefore this output volume fraction was set as a target and performed its prediction through ML models. Following simulations, the ML approach was used not only to improve the performance of the trifurcation microchannel microfluidics devices, but also to expedite the optimization process. In that direction, more than 100 simulations were performed, and that data set was successfully utilized to train the ML models. The three input features considered are inflow velocity, Reynolds number, and the input volume fraction of blood. The outputs include the blood flow rate in the main channel, the maximum output velocity (V_max_), and the maximum volume fraction (Ø_max_) within the channel. The inbuilt library “sklearn.model_selection import train_test_split” was used to train and test these ML models’ performances. This library can randomly split the complete data set into training and testing data sets. Out of the total data sets, 80% data were used to train the ML models, and 20% data (which was unseen by models) set was used for testing purposes. Initially, to understand the correlation between input and output parameters, the correlation plots are plotted as shown in Figure 4.

Correlation plots can help determine whether the three-input feature (Reynolds number, Re, inflow velocity, and inlet volume fraction) have little to no correlation with the target variable (*ϕmax*). This analysis can indicate which of these features may be useful for predictive modeling.

After illustrating the correlation plot, the output feature (*ϕmax*) is predicted by using eight different ML algorithms. The prediction process began with the application of a simple Linear Regression (LR) model, which is a type of supervised learning algorithm [33]. Linear Regression is precisely represented by the equation *y* = β0 + β1*x* + *ε*. Here, y is the dependent variable (ϕ_max_), and x is the independent variable (v in m/s). This model aims to establish a linear relationship between the input features, such as inflow velocity, and the output, which in this case is the volume fraction. Subsequently, Support Vector Machine Regression (SVR) establishes a hyperplane [34] that optimally fits the Ømax data points.

Following SVR, various tree-based machine learning (ML) models such as Decision Trees (DT), characterized by a set of if–else conditions, were investigated for Ømax prediction. The Random Forest (RF) model helps correct the inherent bias of decision trees toward overfitting to the input data points [35]. Adaptive Boosting (AdaBoost) and Gradient Boost (GB) are aimed at enhancing weak learners’ performance. The k-nearest Neighbor(KNN) algorithm, a simple yet robust approach, showcased an impressive R-squared value of 0.97 for output prediction. Finally, Artificial Neural Networks (ANNs), which operate as layered networks, adjusting weights and biases iteratively during training to minimize the output variance, were employed for Ømax prediction.

The performance of these eight different ML models was evaluated using different error metrics, which include Mean Absolute Error (MAE), Mean Squared Error (MSE), Root Mean Squared Error (RMSE), and R^2^ score (Coefficient of determination) [36].

The high-performing ML model shows minimal error values and a maximized R-squared score. The Mean Absolute Error (MAE) calculates the average absolute difference between the predicted and actual values. The Mean Squared Error (MSE), measures the average squared difference between actual and predicted values. Since the unit of MSE is the square of the output column, it can result in larger numerical values that may confuse. This issue is addressed by the Root Mean Squared Error (RMSE) which, like the output column, is expressed in the same unit, providing a more interpretable metric. The R-squared (R^2^) value, also known as the goodness of fit, ranges from zero to one, and its value should be as close to one as possible, indicating good model performance. In regression analysis, relying on a single metric for performance evaluation can be challenging. Therefore, to derive robust conclusions, all four critical performance metrics (MAE, MSE, RMSE, and R^2^) are computed [37]. The relevant Performance Evaluation Metrics can be represented mathematically as expressed below in four equations:

$$MAE=\frac{\sum_{\dot{l}=1}^{N}\left|y_{p}-y_{a}\right|}{N}$$

$$MSE=\sum_{i=1}^{N}{\left(y_{p}-y_{a}\right)}^{2}$$

$$RMSE=\sqrt{\frac{\sum_{i=1}^{N}{\left(y_{p}-y_{a}\right)}^{2}}{N}}$$

$$\mathrm{CO}D=1-\frac{\sum_{i=l}^{N}{\left(y_{p}-y_{a}\right)}^{2}}{\sum_{i=1}^{N}{\left(y_{P}-y\right)}^{2}}$$

where *y_p_* is the predicted number, *y_a_* is an actual number, N is the total sample size, and “*y*” in the COD equation gives an average of actual observations [35].

## 4. Results and Discussion

**Figure 5:**
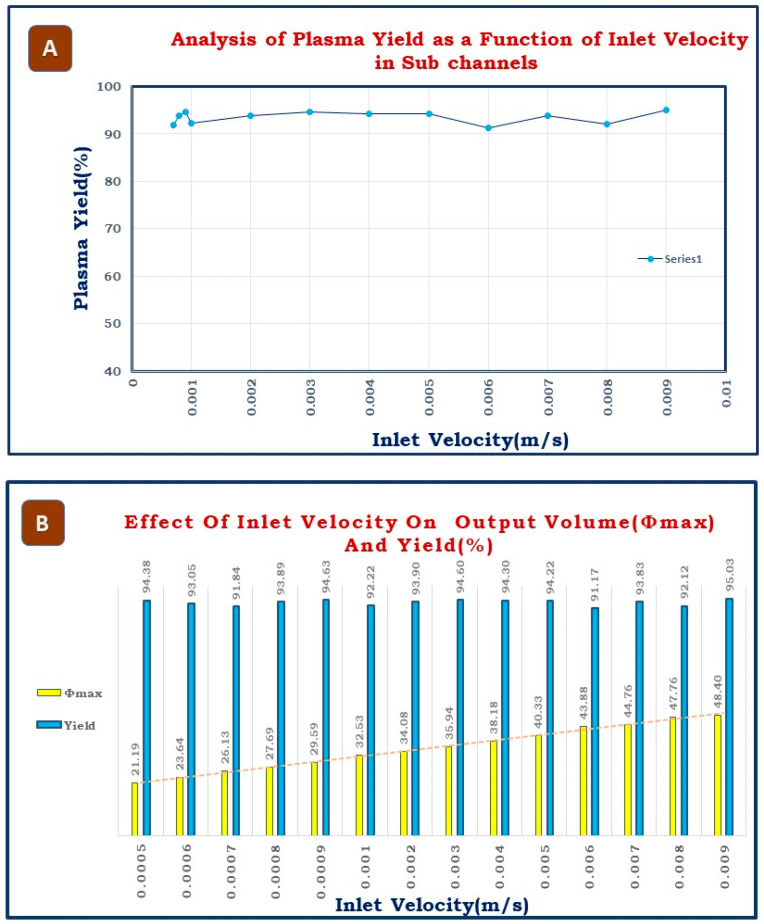
(**A**) Graph depicting the effect of inlet velocity on plasma yield. (**B**) Effect of inlet velocity both on out volume fraction and plasma yield percentage.

The developed microfluidic platform is for the design parameter optimization of the microchannel to separate blood plasma from whole blood. The separation capacity of the microchannel is assessed by determining the plasma yield (%). Since the plasma yield indicates the plasma purity, it was calculated as a percentage using the volume fraction of the dispersed phase at the input and the maximum volume fraction *Ømax* obtained during simulation. During simulations, the input volume fraction values of the dispersed phase ranged from 15 to 46%, resulting in a plasma yield typically between 90 and 95%. Figure 5A illustrates the relationship between plasma yield and inflow velocity. This high yield, reaching approximately 95%, serves as a valuable benchmark for optimizing the design parameters of microchannel for blood plasma separation. Figure 5B demonstrates the effect of inlet velocity on output volume (*Φmax*) and yield. As the inlet velocity increases from 0.0005 to 0.009 m/s, the output volume (*Φmax*) steadily increases, starting at 21.19 and reaching 48.40, indicating a clear positive correlation. In contrast, the yield remains consistently high and stable, fluctuating slightly between 91.17 and 95.03 across all inlet velocities. This suggests that increasing the inlet velocity enhances the output volume fraction *Ømax* without compromising the yield, highlighting an efficient system for achieving higher outputs.

The current study addresses the trade-offs between yield, purity, and processing time in plasma separation using microchannels. Yield refers to the percentage of plasma successfully separated from whole blood, with higher yield being desirable for maximizing usable plasma, while purity indicates the absence of red blood cells (RBCs) and other contaminants essential for diagnostic accuracy. Achieving both high yield and purity is challenging, as increasing inflow velocity can enhance yield, but may reduce purity, while reducing velocity may improve purity, but lower yield. Processing time is inversely related to inflow velocity—higher speeds reduce processing time, but can disrupt separation, leading to purity loss. Our simulations demonstrated a yield of 90–95% without significant purity loss, with an average processing time of 1–3 min, faster than traditional centrifugation. However, there may be slight compromises in purity at higher velocities, which we acknowledge. Future optimization could involve real-time feedback or adaptive machine learning models to refine these trade-offs further.

**Figure 6:**
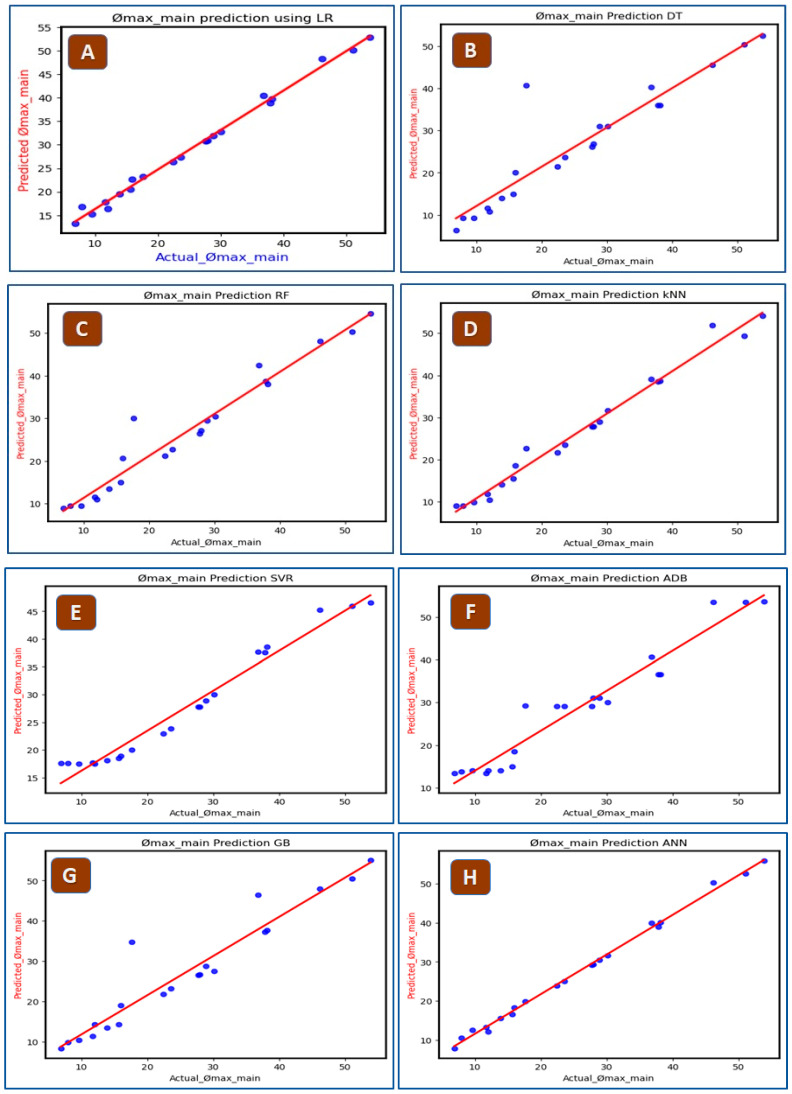
Prediction of output volume fraction with different ML algorithms: (**A**) Linear Regression, (**B**) Decision Tree, (**C**) Random Forest, (**D**) KNN Model, (**E**) SVR Model, (**F**) ADB Model (**G**) Gradient Boost, and (**H**) ANN Model.

After comparing the output volume fraction *Ømax* with plasma yield, the data are trained and tested to give predicted volume fraction *Ømax* with all eight ML models. Figure 6 illustrates the comparison between actual and predicted Ømax values. Also, Table 2 presents a comparative analysis of eight different models used for prediction, focusing on their accuracy metrics in predicting output volume fraction. Among these models, the LR model showed the highest Mean Absolute Error (MAE) at 4.0, whereas the KNN model displayed the lowest MAE at 1.30. Notably, both the k-nearest Neighbor (KNN) and Artificial Neural Networks (ANN) models exhibited the lowest MAE, Mean Squared Error (MSE), and Root Mean Squared Error (RMSE), indicating their superior predictive accuracy. Furthermore, KNN and ANN achieved the highest R^2^ score, suggesting a better model fit compared to the other algorithms. The details of ANN architecture are:

1. Architecture details:

The Artificial Neural Network (ANN) for predicting the output volume fraction (Φmax) was built using default settings from the Scikit-learn library. The network included a single hidden layer with an automatically selected number of neurons, and used the Rectified Linear Unit (ReLU) activation function to introduce non-linearity.

2. Training parameters:

The model employed Stochastic Gradient Descent (SGD) as the default optimization algorithm, with Mean Squared Error (MSE) as the loss function. The default learning rate of 0.001 was applied alongside the default number of epochs and batch size, which adapt dynamically to the data set size and convergence criteria.

3. Justification of default settings:

The default settings were chosen for their simplicity and balance between performance and model complexity. The model achieved an R^2^ of 0.97 and a Mean Absolute Error (MAE) of 1.82, indicating that the default configuration was effective for the task and provided reliable predictions without overfitting.

To validate the simulation outcomes, Table 3 presents a comparison between the simulated output volume fraction (*Ømax*) and the values predicted by the ML algorithms (KNN, ANN). The data presented in Table 3 were generated through an independent simulation process carried out after the training of the ML models. This method ensures that the model predictions were validated using fresh, unseen data, confirming the models’ robustness and ability to generalize in predicting plasma separation efficiency.

Actual (Simulation) *Ømax* represents the actual maximum volume in the microchannel, observed during the simulations for each inflow velocity condition. The flow rate is calculated separately for each value of inflow velocity. The plasma yield percentages achieved under each respective inflow velocity condition were in the range of 93.10% to 95.90%. This data set is valuable for analyzing the performance of predictive models (KNN and ANN). It demonstrates how well these models approximate the actual *Ømax* values obtained from simulations, providing insights into their accuracy and applicability.

Thus, comparing different ML algorithms, the accuracy parameter for designing plasma separation microchannel will be predicted. This process offers a rapid design solution that was previously unexplored for separation channels. Table 4 presents a comparison between prior blood plasma separation studies and the current work, highlighting that this is the first instance of integrating machine learning into the design process. Additionally, the optimization parameter considered in most of the work is the bifurcation angle, whereas in this study, it is the inflow velocity.

## 5. Conclusions

Blood plasma separation is a cornerstone of clinical diagnostics, yet traditional methods remain inefficient and inaccessible for many real-world applications. This study demonstrates a novel ML-integrated microfluidic platform that significantly advances plasma separation technology. By combining CFD-based microchannel simulations with high-accuracy ML predictions, the platform achieves rapid and efficient plasma separation with yields of 90–95%. The trifurcation microchannel design optimizes blood flow dynamics, leveraging the Zweifach–Fung principle to separate plasma and PCV efficiently. ML models, particularly ANN and KNN, exhibited the highest R-squared values (0.97) and lowest error scores (1.30, 1.82) when predicting volume fractions. It enhances the design process by accurately predicting key performance metrics, reducing reliance on iterative prototyping. Furthermore, the integration of computational efficiency in our approach—with simulation times averaging 1–3 min—demonstrates the practical advantages of combining ML and CFD for microfluidic device optimization. This work also highlights the trade-offs between yield, purity, and processing time, addressing these challenges through a balanced approach that maintains high performance across all parameters. The inclusion of computational efficiency in the comparative analysis underscores the superior adaptability and scalability of our platform. By enabling faster plasma extraction and improving healthcare accessibility, the proposed platform holds significant potential to revolutionize clinical workflows, especially in point-of-care and resource-constrained settings. This innovation sets the stage for developing next-generation, portable diagnostic tools tailored to diverse healthcare needs.

## Figures and Tables

**Table 1:** The details of input fluid parameters utilized in the simulation model.

Property	Variable	Expression	Unit
Whole Blood Density	rho (*ρ*)	1057	kg/m^3^
Density of continuous phase (Plasma)	rho (*ρ_c_*)	1000	kg/m^3^
Density of dispersed phase, Packed cell (RBC)	rho (*ρ_d_*)	1095	kg/m^3^
Dynamic viscosity of continuous phase	*µ_c_*	0.003	Pa.s
Fluid consistency coefficient	*µ*_0_	0.02	Pa.s
Flow behavior index	*n*	0.7	1
Apparent viscosity	µ_app_	0.003	Pas
Outlet pressure	p	0	Pa
Reference pressure	P_ref_	1	Pa
Reference temperature	T_ref_	293.15	K

**Table 2:** Prediction for maximum volume fraction (*Ømax*) using ML algorithms.

ML Models	Regression Accuracy Metrics			
	MAE	MSE	RMSE	R^2^ Score
LR	4.00	20.33	4.50	0.89
DT	2.29	27.93	5.28	0.85
RF	1.82	10.73	3.27	0.94
KNN	1.30	4.07	2.01	0.97
SVR	3.24	21.87	4.67	0.88
AdaBoost	3.39	19.86	4.45	0.89
GB	2.35	20.21	4.49	0.89
ANN	1.82	4.06	2.01	0.97

**Table 3:** Comparisons between actual and predicted Ømax values.

Input Inflow Velocities (m/s)	Flow Rate (m^3^/s)	Plasma Yield (%)	Actual (Simulated) *Ømax*	*Ømax* Predicted with KNN	*Ømax* Predicted with ANN
0.0005	1.5 × 10^−8^	93.10	10.74	10.41	10.4
0.001	3 × 10^−8^	93.27	23.61	22.90	22.90
0.019	5.7 × 10^−7^	95.25	33.71	32.5	32.69
0.03	9 × 10^−7^	95.4	40.42	39.00	39.20
0.041	1.2 × 10^−6^	95.90	46.17	44.78	44.80

**Table 4:** Comparative study of microchannel design optimization for blood plasma separation.

References	Separation Technic Adapted	Plasma Yield/ Separati-On Efficiency (SE)	Optimization Method Used	Computational Efficiency	ML Methods Implementation
[17]	Y-junction microchannel with a three-dimensional computational algorithm, VECAM	NA	Microchannel optimization with different bifurcation angles (30° to 180°)	Simulation time not reported.	No ML methods implemented
[19]	Blood flow in tubes, constrictions, and bifurcations, with mixture model using the Navier–Stokes equations and a diffusive flux model (DFM)	Modeling RBC Behavior	Continuum-based approach to simulate red blood cell (RBC) transport in small arteries	Continuum model minimizes the computational grid’s complexity and size.	-
[30]	Separation was modeled using the CFD method based on transfer phenomena. The CFD model with Fick’s law, Navier–Stokes equations, and the Laplace equation.	SE = 99%		Separation time of 12 min.	-
[38]	Zweifach-Fung bifurcation law and constriction expansion. Simulations in COMSOL (mixture model)	yield = 97.03%	Optimization is performed with different Bifurcation angles (30°, 45°, 60°)	Yield is enhanced by reducing the bifurcation angle, narrowing the constriction width, and increasing the number of bifurcating stages.	-
[39]	Multi-branched chip with Zweifach– Fung bifurcation Law and Numerical simulation with SDPD-IBM	SE = 64% yield = 100%	Different branch angles (30°, 45°, 60°, 90°, 180°)	a time-saving and cost-reducing numerical technique with an optimal inflow rate of 13.3 μL/h.	-
[1]	Serpentine separator and constriction–expansion bifurcated Outlet. Simulations COMSOL (Euler-Euler Model)	yield = 98%	-	particle inertial immigration and Euler-Euler model with high throughput but complexity.	-
This work	Zweifach-Fung bifurcation law (Trifurcation channel) Software simulations of 100 microchannels in COMSOL (mixture model)	yield = 97%	Inflow velocities (0.0001–0.01 m/s)	Efficient: Simulation time for each microchannel averaged 1–3 min; low computational resource usage.	Design parameter prediction through ML

## Data Availability

The original contributions presented in the study are included in the article, further inquiries can be directed to the corresponding authors.
